# Learned self-regulation of the lesioned brain with epidural electrocorticography

**DOI:** 10.3389/fnbeh.2014.00429

**Published:** 2014-12-09

**Authors:** Alireza Gharabaghi, Georgios Naros, Fatemeh Khademi, Jessica Jesser, Martin Spüler, Armin Walter, Martin Bogdan, Wolfgang Rosenstiel, Niels Birbaumer

**Affiliations:** ^1^Division of Functional and Restorative Neurosurgery and Division of Translational Neurosurgery, Department of Neurosurgery, Eberhard Karls University TuebingenTuebingen, Germany; ^2^Neuroprosthetics Research Group, Werner Reichardt Centre for Integrative Neuroscience, Eberhard Karls University TuebingenTuebingen, Germany; ^3^Department of Computer Engineering, Wilhelm-Schickard Institute for Computer Science, Eberhard Karls University TuebingenTuebingen, Germany; ^4^Department of Computer Engineering, University of LeipzigLeipzig, Germany; ^5^Institute for Medical Psychology and Behavioural Neurobiology, Eberhard Karls University TuebingenTuebingen, Germany; ^6^Ospedale San Camillo, IRCCSVenice, Italy; ^7^DZD, Eberhard Karls University TuebingenTuebingen, Germany

**Keywords:** electrocorticography, neuroprosthetics, epidural implant, brain-machine interface, neurofeedback, cortical lesion, stroke

## Abstract

**Introduction**: Different techniques for neurofeedback of voluntary brain activations are currently being explored for clinical application in brain disorders. One of the most frequently used approaches is the self-regulation of oscillatory signals recorded with electroencephalography (EEG). Many patients are, however, unable to achieve sufficient voluntary control of brain activity. This could be due to the specific anatomical and physiological changes of the patient’s brain after the lesion, as well as to methodological issues related to the technique chosen for recording brain signals.

**Methods**: A patient with an extended ischemic lesion of the cortex did not gain volitional control of sensorimotor oscillations when using a standard EEG-based approach. We provided him with neurofeedback of his brain activity from the epidural space by electrocorticography (ECoG).

**Results**: Ipsilesional epidural recordings of field potentials facilitated self-regulation of brain oscillations in an online closed-loop paradigm and allowed reliable neurofeedback training for a period of 4 weeks.

**Conclusion**: Epidural implants may decode and train brain activity even when the cortical physiology is distorted following severe brain injury. Such practice would allow for reinforcement learning of preserved neural networks and may well provide restorative tools for those patients who are severely afflicted.

## Introduction

Specific feedback and reward of brain activity allows learning of self-regulation strategies. Operant conditioning of electroencephalography (EEG) and of blood-oxygen-level-dependent (BOLD) signal activity has been applied to reduce disorder-specific symptoms in a variety of neurological and neuropsychiatric conditions (Wyckhoff and Birbaumer, 2014). When neurofeedback is coupled to external devices such as brain-machine interfaces (BMI), the volitional control of brain activity can often be attained, opening up novel training opportunities for the very severely brain-injured and even paralyzed (Buch et al., 2008, 2012; Ang et al., 2011, 2014; Gomez-Rodriguez et al., 2011; Ramos-Murguialday et al., 2012, 2013); first results using EEG-based BMI were promising (Ang et al., 2011, 2014; Ramos-Murguialday et al., 2013). Some -even healthy- participants, however, fail to achieve volitional control of brain activity (Vidaurre and Blankertz, 2010) because of subject-specific anatomical (Halder et al., 2011; Buch et al., 2012; Várkuti et al., 2013) and physiological (Blankertz et al., 2010; Grosse-Wentrup et al., 2011; Vukelić et al., 2014) limitations of the brain, or methodological issues of brain signal recording (Leuthardt et al., 2009). In the context of rehabilitation, additional neurophysiological considerations might contribute to limitations of EEG-based BMI: previous approaches have chosen those frequency bands and algorithms which differentiated best between “motor imagery” and “rest”, e.g., the mu/alpha-band and/or modified common spatial filter algorithms to optimize the selection of temporo-spatial discriminative EEG characteristics (Buch et al., 2008, 2012; Ang et al., 2011, 2014; Ramos-Murguialday et al., 2013). Although even larger groups of stroke patients have participated in BMI training with this approach, a more restricted feature space, e.g., perturbations in the beta-band over selected sensorimotor electrode contacts, might be preferred as a reinforced therapeutic target for restorative purposes (Gharabaghi et al., 2014a,b), despite the fact that they might be less optimal from classification purposes, e.g., to differentiate movement-related brain states in stroke patients (Gomez-Rodriguez et al., 2011; Rossiter et al., 2014).

In general, EEG-based approaches have a characteristically low spatial resolution and a low signal-to-noise ratio because of signal attenuation caused by the skull, possible contamination by muscle artifacts and external electrical activity. These approaches might therefore be specifically challenged in cases of an intentionally limited feature space due to therapeutic purposes. Moreover, they often require a relatively long period of training before subjects can gain real-time control of devices (Birbaumer et al., 1999; Leuthardt et al., 2009; Gharabaghi et al., 2014b).

By contrast, electrocorticographic (ECoG) neurofeedback approaches may be able to surmount such difficulties thanks to their proximity to the neural signal source. We recently proposed a new approach which is less invasive than the classical implanted approaches with *subdural* grids (Yanagisawa et al., 2011, 2012; Wang et al., 2013) or even brain penetrating electrodes (Hochberg et al., 2012; Collinger et al., 2013). This novel approach entailed the application of *epidural* ECoG to decode volitional brain activity in patients with locked-in syndrome suffering from amyotrophic lateral sclerosis (Bensch et al., 2014), with chronic pain as a result of upper limb amputation (Gharabaghi et al., 2014c), and with hemiparesis following subcortical hemorrhagic stroke (Gharabaghi et al., 2014b). In all of these cases, however, most of the cortical tissue of the patients was preserved.

Essential questions with regard to the clinical usefulness of implantable brain-computer interfaces based on epidural ECoG remain unanswered. For instance, would this technique also be applicable in patients with extended cortical lesions? Are these patients able to learn consistent online-control of brain activity? Would high intensity neurofeedback training in these patients be possible? Would ECoG neurofeedback be applicable in patients who are not using volitional control of their brain oscillations with a standard EEG-based approach?

We therefore investigated a brain-machine interface based on epidural ECoG and examined its practicability for neurofeedback training in a patient with an extended ischemic lesion of motor cortical areas who did otherwise not adequately engage in voluntary modulation of brain activity based on EEG recordings.

## Methods

### Patient

**Figure 1:**
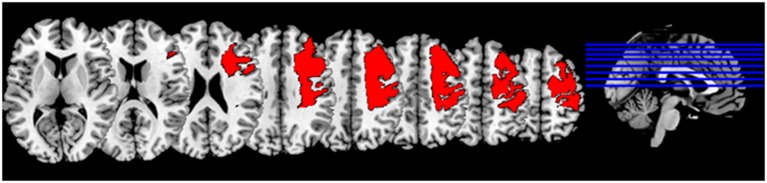
**Lesion mask: Normalized lesion mask displayed on MNI (Montreal neurological institute) brain in standard space (Fonov et al., 2009)**.

The patient, a 52-year-old man, had suffered an ischemic stroke of the right hemisphere with extended cortical lesions (see Figure 1) 13 years prior to implantation. This caused a persistent severe hemiparesis and he no longer had control of his left upper extremity (Medical Research Council motor scale < 2).

**Figure 2:**
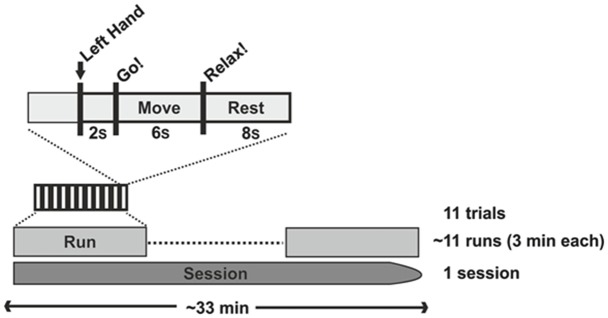
**Study design**.

Several months before surgery, the patient underwent twenty sessions of EEG-based BMI neurofeedback similar to the training described earlier (Ramos-Murguialday et al., 2012; Vukelić et al., 2014) with the same study design that was later used for ECoG-based BMI neurofeedback (see Section Experimental Procedure and Figure 2). Offline evaluation of the EEG data revealed artifacts in the recorded brain signals induced by muscle contraction, i.e., showing EEG amplitudes which exceeded the mean cortical activity by at least two standard deviations. For each feedback electrode (FC4, C4 and CP4) we calculated, separately for the “move” and “rest” period of each trial, the percentage of artifacted samples per session and compared their evolution over time with the respective BMI performance evaluated by the area under the recipient operating characteristics curve (AUC).

Several months later, the patient participated in a different, long-term study for motor cortex stimulation with epidural implants simultaneously with rehabilitation training to improve upper limb motor function following the stroke. The study protocol, approved by the ethics committee of the Medical Faculty of the University of Tuebingen, also involved a four-week evaluation period immediately subsequent to implantation, with electrodes externalized with percutaneous extensions to assess the patient’s cortical physiology for optimization of stimulation. The data shown below is derived from this period.

Following implantation of the electrode array, i.e., several months after the preoperative evaluation with EEG, the patient was subjected to several different experiments for parameter selection and optimization of motor cortex stimulation (not part of the present report) which included altogether 30 ECoG-based neurofeedback sessions with a mean of ~108 feedback trials per session. Due to their heterogeneity these sessions are not suited to evaluate the evolution of BMI performance during this period, however, they may serve as a valuable source of information for estimating the influence of muscle artifacts, which were visually detected during offline analysis, and the feasibility and reliability of ECoG-based neurofeedback.

### Epidural electrocorticography

The epidurally implanted 4 × 4 electrode array consisted of four electrode leads for chronic application (Resume II, Medtronic, Minneapolis, USA) with four platinum iridium electrode contacts, each (4 mm diameter, 10 mm center-to-center distance) covering parts of the right primary motor, somatosensory cortex and premotor cortex. During the evaluation period, the electrode grid was externalized with percutaneous extensions which were connected to a recording and processing unit and a robotic hand orthosis. A monopolar amplifier (BrainAmp MR plus, BrainProducts, Munich, Germany) with 1 kHz sampling rate and a high-pass filter (cutoff frequency at 0.16 Hz) and a low-pass filter (cutoff frequency at 1000 Hz) was used for ECoG recording. Online processing of brain signals was performed using the BCI 2000 framework (Schalk et al., 2004) extended with custom-built features to control an electromechanical hand orthosis (Amadeo, Tyromotion GmbH, Graz, Austria). The data was collected batch-wise, i.e., every 40 ms, the recording computer received a batch of data that contained 40 samples per channel (Walter et al., 2012; Gharabaghi et al., 2014a). The reference electrode was chosen from the contacts on the somato-sensory cortex, i.e., medio-posterior or latero-posterior corner of the grid.

### Experimental procedure

**Figure 3:**
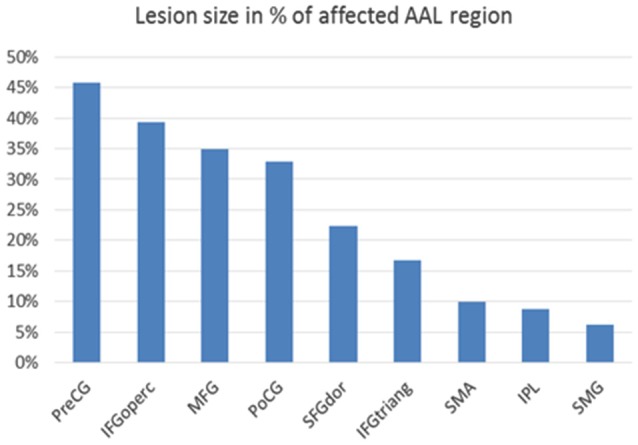
**Lesion size in percentage of affected cortical AAL (=automated anatomical labeling) region (Tzourio-Mazoyer et al., 2002): Affected cortical regions are named according to the AAL brain atlas labels: PreCG = precentral gyrus, IFGoperc = pars opercularis of inferior frontal gyrus, MFG = middle frontal gyrus, PoCG = postcentral gyrus, SFGdor = superior frontal gyrus, dorsolateral, IFGtriang = pars triangularis of inferior frontal gyrus, SMA = supplementary motor area, IPL = inferior parietal lobule, SMG = supramarginal gyrus**.

We used closed-loop, orthosis-assisted opening of the paralyzed left hand which was triggered online by ipsilesional oscillatory brain activity during cued kinesthetic motor imagery of hand opening (Walter et al., 2012; Gharabaghi et al., 2014a). Each session contained 4–16 runs (average 10.86 ± 4.5 runs). Each of the runs had a duration of circa 3 min and consisted of 11 trials. Each trial began with a preparation phase of 2 s, followed by a 6 s movement imagination phase and an 8 s rest phase (see Figure 3). Preparation, imagination and rest phases were instigated by a recorded female voice that gave the commands “left hand”, “go” and “rest” respectively.

A hand orthosis passively opened the affixed left hand as soon as motor imagery-related event-related desynchronization (ERD) in the beta-band (17–23 Hz) was identified during the movement imagination phase. An epoch was regarded as ERD-positive only when the output of the classifier exceeded a threshold. The latter and the electrode selection were determined individually from three training runs before the test sessions (Walter et al., 2012; Gharabaghi et al., 2014a). The spectral power was calculated using an autoregressive model with an order of 16 (McFarland and Wolpaw, 2008) over a normalized 500 ms sliding window shifting every 40 ms. In order to sidestep a noisy control signal for the orthosis, i.e., giving robust and harmonic feedback, we initiated or discontinued orthosis-assisted movement only when five consecutive 40 ms epochs (i.e., 200 ms) where classified as ERD-positive or negative, respectively.

### Performance evaluation

To determine the patient’s ability to modulate his brain activity contingent on the BMI feedback task, we determined the percentage of trials with orthosis movement (i.e., ERD) and the average time with orthosis movement (i.e., ERD) divided by the total feedback duration phase (Gharabaghi et al., 2014b,c).

We also measured a baseline condition to supervise spontaneous perturbations of brain activity which could cause fluctuations in the online performance during the feedback task, i.e., could start the orthosis movement independent of motor-imagery. This baseline condition entailed several ECoG recordings which were taken while the patient rested, i.e., one run with eyes open and one run with eyes closed before each session throughout the whole study period. All in all, we recorded approximately 20 min of such spontaneous baseline ECoG activity for offline analysis, segmented it into trials of the same structure and processed it in the same way as in the feedback sessions (Gharabaghi et al., 2014b,c). For statistical analysis, we used the Matlab toolbox (Wilcoxon rank-sum test) to compare the distribution of performance values per run in each feedback session with the distribution of performance values for the baseline data.

### Imaging evaluation

Before implantation magnetic resonance imaging (MRI) was performed on a 3.0-Tesla Siemens Trio Scanner (TR 1.95 s, TE 2.26 ms, 176 slices of 1 mm slice thickness). For lesion segmentation MRIcron software^1^ was used to manually delineate the lesion. The anatomical image and the mask were normalized to MNI space using SPM 8 (Statistical Parametric Mapping, The Wellcome Department of Imaging Neuroscience, Institute of Neurology, University College London, UK). The overlap of the Automated Anatomical Labeling (AAL) atlas regions and the normalized lesion mask were calculated.

## Results

Lesion segmentation revealed that extended parts of the right hemisphere were affected by the stroke, in particular the primary motor and somatosensory cortex with 45% and 33% lesion size and higher motor areas with 35% (middle frontal gyrus) and 22% (superior frontal gyrus) lesion size with respect to the AAL atlas. The basal ganglia were not affected by the lesion (Figure 3).

**Figure 4:**
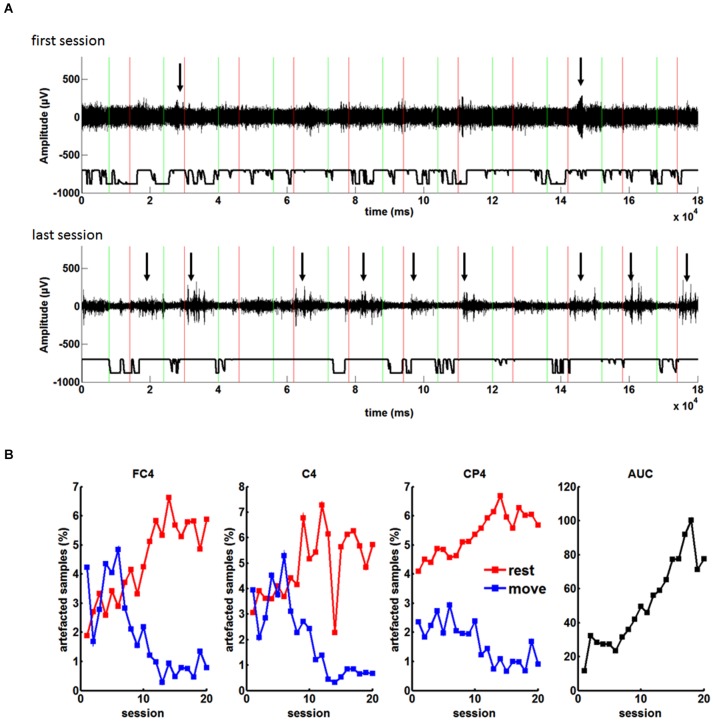
**(A)** EEG recordings during the feedback task with the orthosis before grid implantation: Green and red lines indicate “Go” and “Rest” cues during each trial, respectively. Arrows highlight muscle artifacts during the run. From the first to the last session the number of the artifacts in the rest period of each trial increased. **(B)** Percentage of artifacted samples during the rest and move condition for the three feedback electrodes (FC4, C4, CP4) in the course of twenty sessions. As a result of the increasing difference of artifacts in the rest and the move condition, there was an increase of BCI control measured by the area under the recipient operating characteristics curve (AUC).

EEG analysis of the non-invasive training showed a systematic change of the number of muscle artifacts. In the course of the training, there was an increase of artifacted samples in the “rest” period of each trial and a decrease in the respective “move” periods. The patient learned to increase and decrease muscle tension in the rest period and in the move period of each trial, respectively (see Figures 4A,B).

These changes correlated significantly (*p* < 0.05) with the BMI performance for all channels and both conditions (rest and move), i.e., channel FC4 *r* = 0.8905 for rest and *r* = −0.8254 for move; channel C4: *r* = 0.7045 for rest and *r* = −0.8447 for move; channel CP4: *r* = 0.8878 for rest and *r* = −0.8386 for move (Pearsons correlation coefficient). As a result of the increasing difference between the rest and move condition, there was an increase of BMI control (see Figure 4B), i.e., the increased baseline activity in “rest” made it easier to reach the desynchronization threshold in the “move” period for controlling the BMI. Thus, the patient did not volitionally control his oscillatory brain activity for the neurofeedback training.

**Figure 5:**
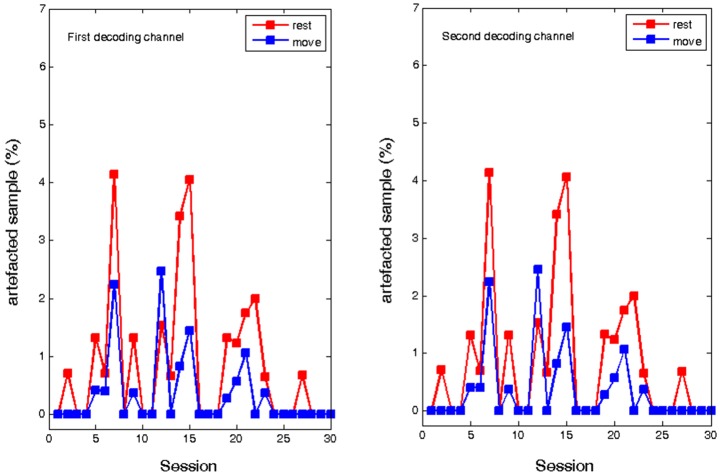
**Percentage of artifacted samples during the rest and move condition of the ECoG recordings for two epidural feedback electrodes in the course of thirty sessions**.

In contrast, ECoG analysis of the implant based training showed no systematic change in the number of muscle artifacts. Due to the low distance of the two recording channels, the number of artifacted samples was identical. In the course of the training, there was a fluctuating amount of artifacted samples both in the “rest” period and in the “move”. Similar to the EEG experiment there were more artifacts in the rest period, but showed no evolution over time. Thus, although muscle tension was not completely eliminated, it did not influence the volitional control of oscillatory brain activity (see Figure 5).

**Figure 6:**
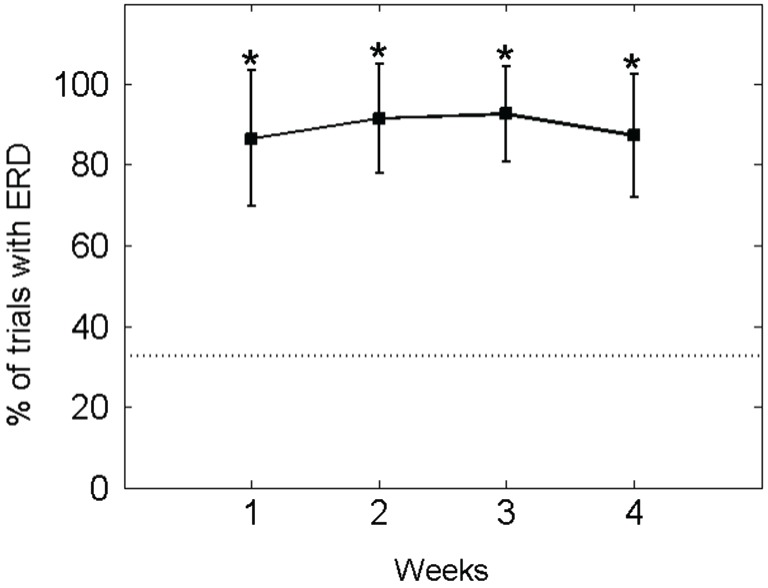
**Percentage of EcoG trials with orthosis movement (i.e., event-related desynchronisation [ERD] in the beta-band): The mean ± standard deviation of the performance measure per week is indicated by solid lines**. The mean of the baseline data is indicated as a dotted line. An asterisk (*) marks weeks in which the mean of the performance measure differs significantly (*p* < 0.05) from the mean of the baseline value.

Accordingly, in the ECoG-based approach, the patient modulated his motor-imagery related ERD contingent on the BMI feedback task, i.e., initiated the orthosis movement in a mean of 90.49 ± 13.73% of all trials (baseline condition: 32.72 ± 9.77%), thus retaining significant control of brain activity throughout the whole study period (see Figure 6).

**Figure 7:**
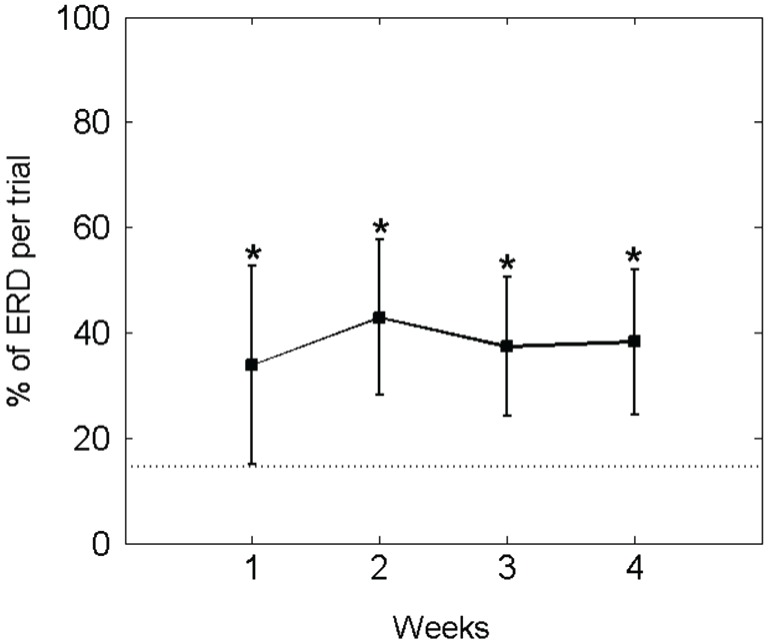
**Percentage of average ECoG-based orthosis movement (i.e., event-related desynchronisation [ERD] in the beta-band) divided by the total feedback duration phase: The mean ± standard deviation of the performance measure per week is indicated by solid lines**. The mean of the baseline data is indicated as a dotted line. An asterisk (*) marks weeks in which the mean of the performance measure differs significantly (*p* < 0.05) from the mean of the baseline value.

In fact, he controlled the orthosis movement (i.e., ERD) for a mean of 37.15 ± 15.27% of the feedback duration in each trial. Thus, his performance in this online closed-loop paradigm was constant and significantly higher than in the baseline condition (14.52 ± 7.30%) throughout the study period (see Figure 7).

## Discussion

The patient presented here—with an extended ischemic lesion of the cortex—learned control of high intensity neurofeedback training based on self-regulation of brain oscillations recorded from the epidural space by ECoG. Although the ECoG based approach enabled the patient to maintain consistent control of his sensorimotor rhythms in the beta-band in an online closed-loop paradigm throughout the study period, his performance in controlling the neurofeedback device in ~30–40% of the feedback duration was—while significantly better than baseline (~15%)—nonetheless markedly lower than comparable ECoG-based (Gharabaghi et al., 2014b) or EEG-based (Ramos-Murguialday et al., 2013) approaches in other similarly affected patients who had attained control rates of ~50–60% of the feedback duration. These variations in performance might be explained by physiological and morphological differences: The respective patients showed strikingly different baseline conditions, i.e., spontaneous perturbations of brain activity in the beta-band could start the orthosis movement independent of motor-imagery during ~15% vs. ~30% of the feedback period in the present and in previous cases (e.g., Gharabaghi et al., 2014b), respectively. These physiological baseline differences could be explained by the different lesion characteristics, namely extended cortical vs. circumscribed subcortical lesions, respectively. Since this brain activity is known to originate from primary motor and somatosensory as well as from secondary motor areas, the most plausible explanation for the decrease of spontaneous perturbations in the presented case is that they have been affected by the lesion. Our results are in line with recent findings that movement-related beta desynchronization in the contralateral primary motor cortex was found to be significantly reduced in stroke patients compared to controls, while within this patient group, smaller desynchronization has been seen in those with more motor impairment (Rossiter et al., 2014). Moreover, these observations support our general strategy, applied in the present case as well, to choose beta-band desynchronisation as a therapeutic target for restorative interventions in severely affected stroke patients (Gharabaghi et al., 2014a,b).

An intriguing insight gained in this study was that the epidural ECoG technique enabled the patient to engage in feedback exercises based on voluntary modulation of brain activity despite the fact that he did otherwise not use properly a standard EEG-based approach. Interestingly enough, prior to using the implanted brain interface, the patient learned to increase and decrease muscle tension in the rest period and in the move period of each trial, respectively, for BMI control. This alternative conditioning probably occurred because the extent of his own voluntary modulation of brain activity was too insignificant to be detected by EEG whereas the muscle contractions could sufficiently be detected and were reinforced by feedback and reward. This alternative control strategy applied by the patient was unexpected. The participants in this study and in previous studies with healthy subjects (Vukelić et al., 2014) and similarly severely affected stroke patients (Ramos-Murguialday et al., 2012) were instructed to avoid blinking, chewing, head and body compensation movements. Along with visual inspection and feedback by an experienced examiner this approach proved to be a sufficient method to prevent alternative BMI control in the past. Moreover, the examiners were prepared to detect compensatory movements *during* the “move” phase of the feedback task as this is the most commonly observed strategy to pretend volitional modulation of ERD, and not *before* the actual task in the “rest” phase. Therefore, increasing baseline activity in “rest” through elevated muscle tension and concurrent reduced muscle tension in the “move” period, have in future to be considered as subtle bypassing strategies to reach the desynchronization threshold more easily.

For this purpose, online detection of EMG contamination with dedicated spectral and topographical analyses might be necessary to prevent alternative BMI control in future. Previous work in this field was conducted without such precautions most probably due to the fact that lower frequency bands were applied for BMI control, which are usually less affected by muscle artifacts (Goncharova et al., 2003). However, due to their relevance for sensorimotor control (Kilavik et al., 2013; Brittain et al., 2014), motor learning (Herrojo Ruiz et al., 2014) and corticospinal excitability (Takemi et al., 2013) as well as due to their correlation with the extent of functional impairments after stroke (Rossiter et al., 2014), higher frequency bands in the beta range might be considered in future more often as therapeutic targets for restorative EEG neurofeedback and motor rehabilitation (Gharabaghi et al., 2014a,b), necessitating the consideration of even subtle EMG contamination as observed in the presented case. EMG artifact detection may include relatively simple methods such as rejection of EEG segments that exceed a predefined amplitude threshold or more sophisticated methods such as factor decomposition using principal component or independent component analysis with or without source reconstruction algorithms (Goncharova et al., 2003; Hipp and Siegel, 2013). In any case, applicable approaches need to work even with only few available channels within a narrow frequency band and have to provide real time processing and low computational complexity (Tiganj et al., 2010).

Should EMG artifacts turn out to be too difficult to mitigate (yet not explicitly addressed by this study) or should the targeted physiological brain state, e.g., motor imagery-related beta-band desynchronisation, be too weak to be robustly detected in the EEG of severely affected stroke patients, implantable approaches might provide an alternative. In this context, the ECoG approach has two advantages over EEG: On account of its proximity to the neural signal source, it surmounts difficulties related to signal attenuation caused by the skull. It is also less susceptible to contamination by muscle artifacts, and, in this case, benefits from the signal attenuation caused by the skull. In this vein, simultaneously recorded ECoG and EEG activity in motor cortical areas revealed that invasively measured signals had a twenty to hundred times better brain signal quality than signals that were acquired non-invasively (Ball et al., 2009).

The technique presented here is limited by the necessity to connect the intracranial implant to an external online processing framework for recording and neurofeedback training via extension leads which are externalized through the skin (Gharabaghi et al., 2014b,c). Future applications of this brain self-regulation approach will require wireless devices capable of fast and reliable information transfer (Borton et al., 2013; Piangerelli et al., 2014). This would facilitate the application of this intervention on a day-patient basis or even in the patient’s home environment.

However, before drawing definite conclusions regarding effectiveness of various neurofeedback approaches, future studies need to directly compare ECoG-based techniques to EEG-based methods which control for EMG artifacts. This research needs to consider further aspects such as direct and indirect costs, complications, learning curve, motivation, applicability for long-term use and the possibility of performing training independent of professional support. Based on the respective findings, patients with different impairment levels might then be referred to the specific treatment modality best suited for the individual pathophysiological state.

In conclusion, epidural implants could provide reliable feedback interfaces for brain self-regulation in patients in whom non-invasive approaches fail on account of signal attenuation caused by the skull or due to the underlying pathophysiology. This could establish them as valuable tools in the context of reinforcement learning in a variety of neurological and neuropsychiatric conditions.

## Conflict of interest statement

The authors declare that the research was conducted in the absence of any commercial or financial relationships that could be construed as a potential conflict of interest.

## Footnotes

^1^http://www.mccauslandcenter.sc.edu/mricro/mricron/install.html
